# Liquid biopsy in pediatric acute lymphoblastic leukemia

**DOI:** 10.3389/fonc.2026.1853549

**Published:** 2026-06-22

**Authors:** Yingcong Chen, Jie Wang, Cuiying Ye, Xudong Xu, Meina Yue, Xinfeng Zhao

**Affiliations:** Department of Clinical Laboratory, Hangzhou Children’s Hospital, Zhejiang, China

**Keywords:** circulating tumor DNA, extracellular vesicles, liquid biopsy, microRNA, pediatric acute lymphoblastic leukemia

## Abstract

Acute lymphoblastic leukemia (ALL) is the most common pediatric malignancy, and despite advances in therapy, relapse remains a major cause of treatment failure. Liquid biopsy has emerged as a powerful, minimally invasive tool for real-time disease monitoring and prognostication in hematologic malignancies. This review summarizes recent progress in the application of liquid biopsy technologies in pediatric ALL, focusing on circulating tumor DNA (ctDNA), circulating microRNAs (miRNAs), and extracellular vesicles (EVs). We discuss their respective biological origins, detection platforms, and clinical utilities in diagnosis, risk stratification, and measurable residual disease (MRD) assessment. Particular attention is paid to pediatric-specific challenges such as limited blood volume, pre-analytical variability, and the need for sensitive assays adapted to the pediatric context. Furthermore, we highlight cutting-edge innovations in EV isolation, machine learning-based biomarker integration, and prospective clinical applications. Notably, bone marrow evaluation remains the irreplaceable gold standard for pediatric ALL diagnosis and MRD monitoring. Although most liquid biopsy approaches are still in early translational stages for pediatric ALL, accumulating evidence supports their complementary value for optimizing individualized patient management. Continued validation in large, prospective pediatric cohorts is essential to bring these technologies closer to clinical implementation.

## 1 Introduction

Acute lymphoblastic leukemia (ALL) is the most common pediatric cancer, arising from malignant proliferation of lymphoid precursors in the bone marrow (1). Cure rates for childhood ALL now approach ~90% in developed countries with risk-adapted therapy (2). However, prognosis remains poor for patients with relapsed ALL, and treatment is intensive with significant toxicities (3). Traditional diagnosis and monitoring of ALL rely on bone marrow aspirates for morphology, immunophenotyping, cytogenetics, and minimal residual disease assessment (3). These invasive procedures pose risks and discomfort, especially for children, and may miss occult disease in sanctuary sites like the central nervous system (CNS) (4). In this context, liquid biopsy – the analysis of tumor-derived biomarkers in body fluids – has emerged as an attractive, non-invasive approach for cancer diagnostics and monitoring (5). Liquid biopsy could enable frequent, low-risk sampling to detect disease burden, prognostic markers, or impending relapse without repeated bone marrow biopsies (5).

**Figure 1:**
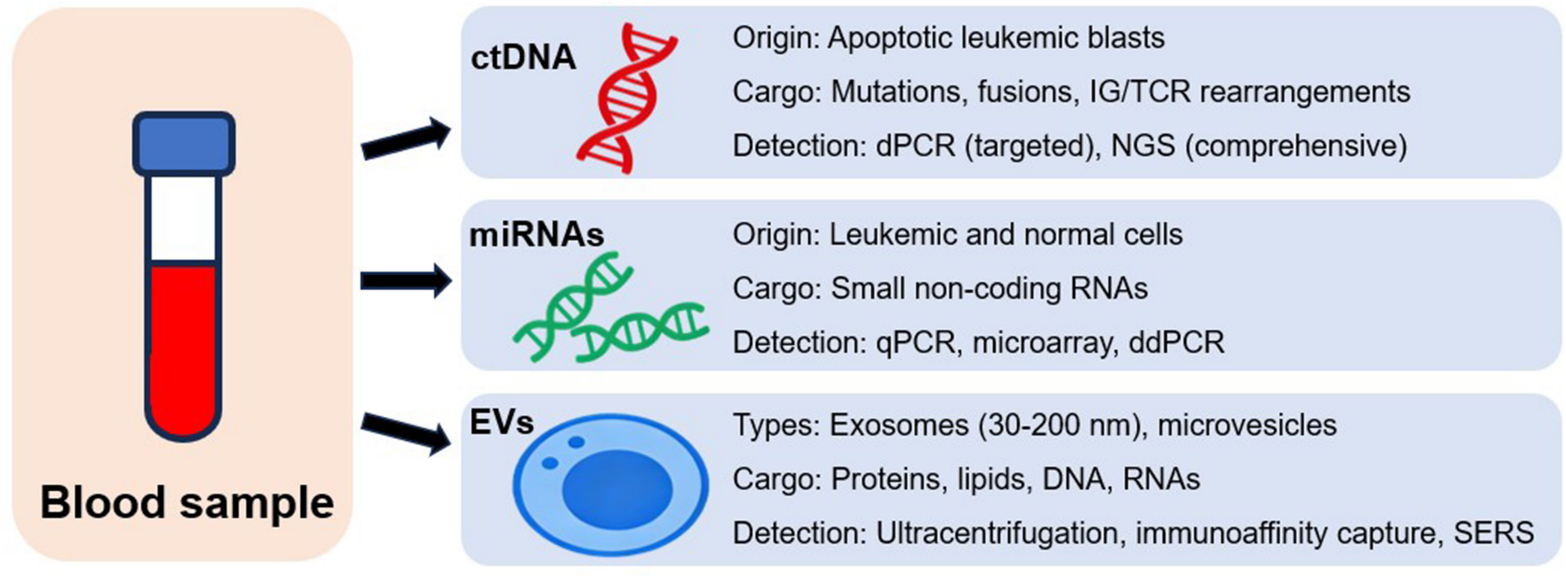
Liquid biopsy biomarkers in pediatric ALL.

This review focuses on recent advances (primarily within the last ~5 years) in liquid biopsy for pediatric ALL. We discuss key circulating biomarkers – circulating tumor DNA (ctDNA), circulating microRNAs (miRNAs), and extracellular vesicles (EVs) (including exosomes) – and their clinical applications in ALL for diagnosis, prognosis, MRD monitoring, and relapse prediction, as shown in **Figure 1**. We outline the strategies for sample collection and detection (e.g. next-generation sequencing and digital PCR platforms) and compare these with traditional bone marrow-based methods. Current challenges unique to pediatrics, such as limited sample volumes, the need for ultra-sensitive assays, standardization issues, and ethical considerations, are examined. While adult oncology has led the way in liquid biopsy implementation, we highlight pediatric-specific progress and the potential of liquid biopsy to complement rather than replace invasive bone marrow monitoring in childhood ALL. We strictly distinguish established routine marrow assays from investigational liquid biopsy approaches throughout this review.

## 2 Liquid biopsy sampling and detection strategies in pediatric ALL

Liquid biopsy in ALL typically involves minimally invasive sampling of blood (peripheral venous blood), from which plasma is isolated as the source of circulating biomarkers. Plasma is preferred over serum, since coagulation in serum can release additional cellular DNA that dilutes the tumor-specific fraction (5). For certain clinical scenarios, other biofluids may be informative – for example, cerebrospinal fluid (CSF) can be analyzed for leukemic biomarkers in cases of CNS leukemia (5). Indeed, liquid biopsy of the CSF is being explored to detect CNS involvement in ALL that might be missed by cytology or flow cytometry of CSF cells (4). Other fluids (like urine or saliva) are not commonly used for ALL, given that leukemia is centered in the marrow and blood; thus, blood-based assays remain the mainstay. Leukemic blasts possess high proliferative activity and spontaneous apoptosis propensity, allowing genomic fragments to be continuously released into peripheral circulation. These inherent biological features make it feasible to trace leukemia-derived genetic material via liquid biopsy approaches.

**Figure 2:**
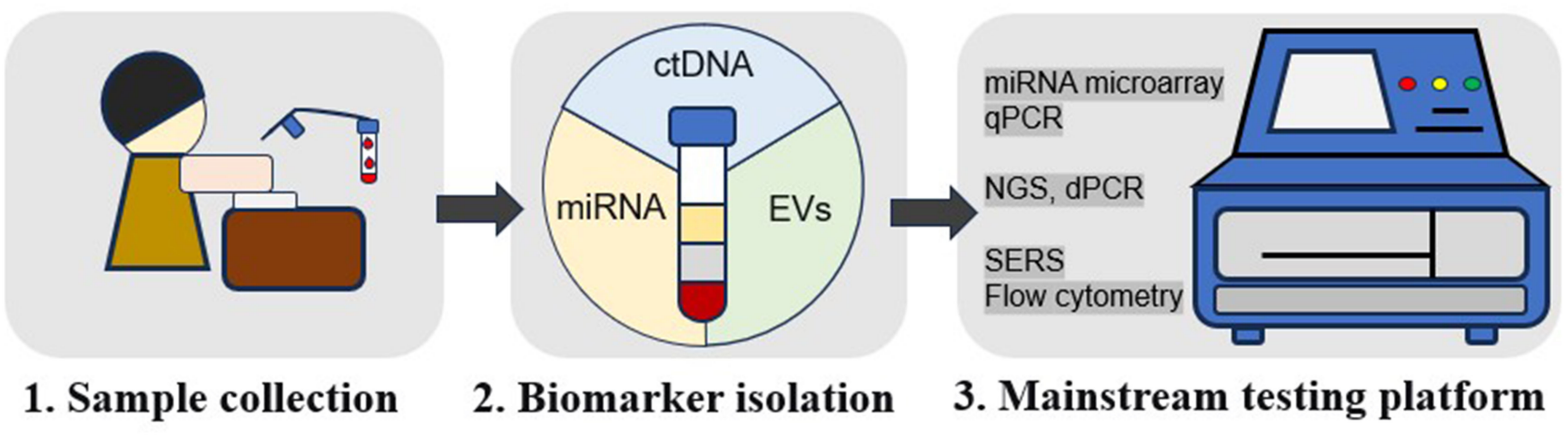
Schematic workflow of liquid biopsy detection in pediatric ALL.

Detection platforms for liquid biopsy have rapidly evolved. Two of the most important technologies are digital PCR (dPCR) and next-generation sequencing (NGS). Digital PCR – including droplet digital PCR (ddPCR) – allows precise quantification of nucleic acids by partitioning the sample and can detect extremely low-level mutations or DNA fragments with high sensitivity (6). It is well suited for targeted detection of known leukemia-associated genetic markers (for example, a specific ALL gene fusion or point mutation) and has been used to quantify *miRNAs* as well (4). NGS, on the other hand, enables broader genomic analysis. Targeted NGS panels or even whole-genome sequencing can identify leukemia-specific sequences in cell-free DNA or RNA without prior knowledge of the exact mutation (7). Modern NGS approaches with error-suppression techniques (unique molecular identifiers, duplex sequencing, etc.) can achieve very high sensitivity for low-frequency variants (8). For instance, error-corrected NGS has been shown to detect mutant sequences down to variant frequencies around 0.01% (8) – critical for MRD applications. Specialized methods have also been developed for ALL, such as high-throughput sequencing of B-cell receptor/IGH gene rearrangements to track the ALL clone (9). Current detection platforms differ substantially in limit of detection, sample input volume, multiplexing capacity and turnaround time, with error-corrected NGS and ddPCR being the most adaptable approaches for low-volume pediatric ALL samples (10, 11). A simple schematic diagram of ALL sample testing based on mainstream detection platforms is shown in **Figure 2**.

Sampling frequency and timing are also important considerations. Liquid biopsy allows repeated sampling during therapy and follow-up with minimal risk (5). For example, blood could be drawn at diagnosis, end of induction, and periodic intervals to monitor molecular response, whereas bone marrow aspirates are typically limited to a few key time points due to invasiveness. Studies in adult lymphoid malignancies suggest that peripheral blood monitoring can sometimes substitute for marrow biopsies for MRD, though more validation is needed in ALL (12). Importantly, liquid biopsy may capture information that a single-site marrow sample misses – reflecting the whole-body disease burden and clonal heterogeneity (5). That said, bone marrow evaluation remains the gold-standard for ALL diagnosis and risk stratification at present, given its well-established sensitivity (flow cytometry or PCR-based MRD down to 10^−4^~10^−5^) and ability to directly visualize leukemic blasts (12). Liquid biopsy assays must achieve comparable sensitivity and reproducibility to replace or augment marrow-based methods.

Comparison with bone marrow: Liquid biopsies are unquestionably less invasive than bone marrow aspirates, reducing pain and anesthesia risks for pediatric patients (5). This aligns with the ethical mandate in pediatrics to minimize risk and discomfort whenever possible (13). Additionally, blood-based tests can be repeated more frequently to closely track disease dynamics. However, challenges include the lower tumor DNA fraction in blood compared to marrow. In remission or early disease, circulating tumor DNA can be exceedingly scarce (often <0.01% of total cell-free DNA) (8), demanding highly sensitive methods to avoid false-negatives (12). By contrast, a marrow aspirate samples the primary tumor compartment, typically yielding a higher proportion of leukemic cells (if any are present). Another consideration is that certain standard assessments – like examining cellular morphology or performing full immunophenotyping – currently require a marrow sample with intact cells; liquid biopsy cannot provide these directly. Thus, rather than outright replacing bone marrow evaluation, liquid biopsy in pediatric ALL is envisioned as a complementary tool that can provide additional molecular insights and enable interim monitoring between marrow evaluations^[12^. Indeed, clinical practice is already trending that way in some adult hematologic malignancies – for instance, ctDNA-based MRD assays are now included in guidelines for diffuse large B-cell lymphoma – and pediatric ALL trials are beginning to incorporate liquid biopsy research endpoints (14, 15). However, such blood-based approaches can only serve as a complementary strategy and cannot replace routine bone marrow examination at present. In the diagnosis of ALL, a comparison between liquid biopsy and bone marrow aspiration is shown in **Table 1**.

**Table 1:** Bone marrow aspirate vs. liquid biopsy in pediatric ALL diagnosis.

Aspect	Bone marrow aspirate	Liquid biopsy
Invasiveness	Highly invasive; requires anesthesia/sedation; painful	Minimally invasive; routine blood draw; well-tolerated
Sampling frequency	Limited to key time points (diagnosis, end of induction)	Repeated sampling allowed for dynamic monitoring
Sample representation	Localized to puncture site; may miss focal/ extramedullary disease	Systemic; reflects whole-body tumor burden and clonal heterogeneity
MRD sensitivity	Gold standard (10^-4^ to 10^-5^ by flow cytometry/PCR)	High sensitivity only under optimized experimental conditions (10^-5^ to 10^-6^; not for routine clinical use)
Information provided	Cellular morphology, immunophenotyping, cytogenetics	Molecular alterations (ctDNA mutations, miRNA profiles, EV cargo)
Pediatric challenges	Procedural risk, pain, psychological trauma	Limited blood volume, need for ultra-sensitive assays
Current role	Essential for diagnosis and key timepoint assessment	Complementary tool for auxiliary monitoring
Core clinical scenarios	Definitive diagnosis, risk stratification, treatment endpoint evaluation	Interim dynamic monitoring, early relapse warning, extramedullary lesion auxiliary assessment

MRD sensitivity varies significantly depending on detection platform, sample quality, disease subtype and biological matrix, and cannot be generalized simply.

## 3 Circulating tumor DNA in pediatric ALL

Circulating tumor DNA refers to the fragmented DNA derived from cancer cells that is released into the bloodstream (as part of the cell-free DNA pool). In leukemia, ctDNA originates from dying leukemic blasts in the bone marrow and, if present, elsewhere. Notably, ctDNA is usually a small fraction of total cell-free DNA (<10% and often much less in remission) (5). Of note, total cell-free DNA (cfDNA) originates from all types of normal somatic cell turnover, while ctDNA specifically refers to the tumor-derived fraction carrying malignant genetic alterations. Despite its low abundance, ctDNA carries the same genetic alterations as the leukemia cells, such as point mutations, gene fusions, copy number changes, or immunoglobulin/T-cell receptor (IG/TCR) gene rearrangements (5). This makes ctDNA a powerful biomarker – essentially a “molecular biopsy” of the leukemia that can be obtained from plasma. Leukemia-derived ctDNA is mainly concentrated in 160-180 bp fragment range, and largely retains the molecular characteristics of primary leukemic mutations. Bioinformatics profiling and variant filtering can effectively distinguish leukemia-specific ctDNA from background normal cell cfDNA, with optimized assays achieving high detection sensitivity under experimental conditions (16, 17). Moreover, intratumoral heterogeneity across distinct leukemic subclones leads to divergent proliferation and spontaneous apoptotic rates, resulting in variable quantities of released ctDNA into peripheral blood and partially accounting for uneven detectability of liquid biopsy biomarkers among individual pediatric ALL patients.

1. Detection of ctDNA: A variety of approaches have been used to detect ctDNA in ALL. If a patient’s leukemia harbors a known recurrent fusion (e.g. *ETV6-RUNX1* or *BCR-ABL1*) or mutation, *targeted* assays (qPCR or ddPCR) can directly measure those in plasma. For example, quantitative PCR for BCR-ABL1 transcripts in blood is routinely done in chronic myeloid leukemia and Philadelphia-positive ALL; similar strategies could apply to other ALL-specific aberrations. More comprehensive methods sequence a broader set of targets: targeted gene panels for ALL mutations, or, uniquely, the immunoglobulin heavy chain (IGH) rearrangement in B-ALL. Each B-ALL has a clone-specific IGH sequence that can serve as a precise marker of that leukemia clone. Researchers have exploited this by deep-sequencing IGH from plasma DNA. Beagan et al. (2021) demonstrated that shallow whole-genome sequencing of plasma cfDNA could identify leukemia-associated copy number changes without PCR bias (18). More recently, Sampathi et al. (2022) developed a Nanopore MinION sequencing workflow to amplify and sequence IGH from plasma and *CSF* of pediatric B-ALL patients (6). This rapid, low-cost approach successfully detected the ALL clonal IGH sequences in all tested patients, including those with CNS involvement (6). The authors could track multiple clones in parallel and found that ctDNA-based IGH monitoring detected persistent disease when conventional flow cytometry MRD was negative, and identified occult CNS leukemia via CSF cfDNA when routine CSF cytology showed no detectable leukemic blasts. Such occult ctDNA positivity triggers upward reallocation into higher-risk subgroups, prompting intensified periodic disease surveillance; in selected high-risk cohorts, clinicians may also consider modified consolidation or maintenance regimens to lower prospective relapse risk. This underscores the ultra-sensitivity of ctDNA assays – potentially exceeding standard methods, since even if leukemic cells are sequestered in the CNS or present below cytometric detection in marrow, their DNA may still be picked up in biofluids (6).

In pediatric ALL, ctDNA detection faces unique biological and technical bottlenecks, including extremely low plasma tumor fraction during clinical remission, short half-life of cfDNA, and strict pediatric blood volume constraints. False negatives are mainly caused by insufficient tumor shedding and blood–brain barrier isolation, while false positives derive from sequencing artifacts and clonal hematopoiesis. There are obvious differences in ctDNA release patterns between B-ALL and T-ALL, and NGS validated performance in bone marrow cannot be directly extrapolated to routine plasma ctDNA monitoring (19).

2. Clinical applications of ctDNA: The primary uses of ctDNA in pediatric ALL lie in treatment monitoring, MRD detection, and relapse prediction. Various studies suggest ctDNA levels correlate with disease burden. For example, at diagnosis (when marrow blasts are high), ALL patients tend to have elevated total cfDNA concentrations, which then decline with therapy. A 2024 study by George et al. found that early changes in cfDNA levels could stratify prognosis in acute leukemias – patients whose cfDNA did not drop appropriately had worse outcomes (20). More specifically, the presence of molecular residual disease in plasma after therapy is emerging as a potent risk factor. In ALL, MRD by bone marrow is the strongest prognostic factor for relapse (12). If ctDNA assays can detect MRD non-invasively, it could revolutionize follow-up. Evidence is building: in the Nanopore IGH study, ctDNA was detectable in patients who were ostensibly MRD-negative by standard tests (6). Another group demonstrated in a patient-derived xenograft model that ctDNA levels mirrored leukemia burden and could be used to monitor treatment response (21). There are also anecdotal reports of ctDNA revealing impending relapse earlier than clinical signs. For instance, an extreme case reported ALL-associated DNA detected in a pregnant woman’s blood during routine prenatal screening (due to fetal cfDNA testing) – this led to the unexpected diagnosis of maternal ALL with an iAMP21 chromosomal abnormality (22). In relapse settings, tracking ctDNA may identify resurgence of the original clone or evolution of therapy-resistant clones. Hollanda et al. (2025) emphasize that ctDNA can be used to track clonal evolution over time in leukemia, capturing new mutations that arise under therapeutic pressure (1, 23). This could inform timely changes in therapy (e.g. adding targeted drugs if a mutation is detected). Such occult ctDNA positivity can assist early risk re-stratification and guide intensified long-term follow-up surveillance for high-risk pediatric ALL patients.

One clear advantage of ctDNA is that it integrates genetic information from all disease sites. Particularly in ALL, it may help detect extramedullary disease. CNS relapse of ALL is a notorious challenge – leukemia may infiltrate the CNS even when bone marrow is in remission, and detection currently relies on lumbar puncture and microscopy, which lack sensitivity (4). Cell-free DNA analysis of CSF or even plasma might serve as a “liquid CNS biopsy.” The above-mentioned nanopore approach and others have indeed found ALL ctDNA in CSF supernatant and plasma that correlates with CNS leukemia (4). For instance, patient-specific ctDNA has been validated as a sensitive biomarker to monitor therapeutic response to menin inhibitors in preclinical models of infant leukemia (21). If MRD-positive ctDNA is found months before a scheduled marrow exam, clinicians could intervene earlier. That said, these applications are still largely investigational in pediatrics. The sensitivity challenge is non-trivial: when a child is in deep remission, only a few dozen leukemia cells may be present in the body (~10^−6 level), and capturing their DNA in a 1–2 mL plasma sample is like finding a needle in a haystack. Techniques like ultra-deep sequencing with error correction or personalized patient-specific assays are required to reach this level (12). Early results are promising, but larger prospective trials are needed to determine how reliably ctDNA MRD predicts relapse in pediatric ALL and how it should guide therapy changes (12).

In summary, ctDNA has emerged as a versatile biomarker in pediatric ALL, with proven utility in identifying genomic features non-invasively and strong potential in MRD and relapse monitoring. It represents a paradigm shift from cell-based assessment to DNA-based assessment of leukemia burden. As assays are refined and standardized, ctDNA could substantially reduce the need for frequent bone marrow aspirates and provide a more continuous readout of disease status during and after therapy. Among all liquid biopsy biomarkers, ctDNA-based detection possesses the most robust clinical rationale and validation evidence.

## 4 Circulating microRNAs in pediatric ALL

MicroRNAs (miRNAs) are small (~22 nucleotide) non-coding RNA molecules that regulate gene expression, often by binding mRNA transcripts. They are released into the circulation by both normal and cancer cells and remain stable in blood, protected from RNases by vesicles or protein complexes. In cancer, dysregulated miRNA profiles reflect the underlying tumor biology, and thus circulating miRNAs have been widely studied as biomarkers in solid tumors and leukemias (2). Pediatric ALL is no exception – numerous studies in the last decade have identified abnormal patterns of circulating miRNAs in children with ALL compared to healthy individuals (24). These miRNA signatures are being explored for diagnostic and prognostic applications.

Several specific miRNAs have emerged as candidates. For example, miR-146a has been regarded as a potential candidate biomarker in ALL. In a 2021 study by Shahid et al., plasma miR-146a was significantly overexpressed in ALL patients at diagnosis (both pediatric and adult cases) compared to healthy controls (2). Notably, miR-146a levels dropped after chemotherapy in those patients who achieved remission (2). This suggests that miR-146a correlates with disease presence and burden. The study concluded that plasma miR-146a may act as a potential non-invasive candidate for auxiliary diagnosis and prognostic assessment in ALL, with higher levels at baseline potentially indicating a worse prognosis (2). Importantly, the miR-146a levels were independent of age, gender, or initial white cell count, and thus might add new information beyond traditional risk factors (2).

Other miRNAs reported in pediatric ALL include miR-155, miR-181a, miR-210, miR-29 and many others, often found to be aberrantly expressed in ALL versus normal controls (24). Recent work by Wang et al. (2024) identified a novel panel of three circulating microRNAs—hsa−miR−27a−5p, hsa−miR−142, and hsa−miR−411−5p—within plasma samples of pediatric ALL patients that displayed stepwise elevation in expression levels, progressing from healthy controls to newly diagnosed patients and peaking in relapsed individuals (25). Utilizing high-throughput microarray technology and subsequent RT−qPCR validation, the combined miRNA panel showed favorable discriminative potential in relapse detection, with an area under the ROC curve (AUC) of 0.89 in the training cohort and 0.95 in matched bone marrow samples. Integrated into a risk-prediction nomogram alongside clinical features (e.g., fusion gene status, day-33 MRD), this miRNA signature may serve as an independent prognostic indicator for relapse, contributing significantly to improved stratification capabilities (overall combined AUC 0.96) (25). The findings underscore the clinical potential of these circulating miRNAs as minimally invasive biomarkers for early relapse prediction in pediatric ALL. Individual miRNAs have also been associated with prognosis or specific ALL subtypes, predominantly pediatric B-ALL. miR-181a is particularly interesting in the context of CNS leukemia. A recent 2024 study by Péterffy et al. used *droplet digital PCR* to measure miR-181a in cell-free CSF from pediatric ALL patients (4). They found that high miR-181a copy number in CSF strongly correlated with CNS leukemia involvement (4). In fact, measuring miR-181a helped stratify patients with otherwise ambiguous CNS status: some children with no blasts seen on cytology had elevated CSF miR-181a, indicating occult CNS disease (4). This could have potential clinical reference value for clinical decision-making, as those patients might benefit from intensified CNS-directed therapy (4). Thus, miR-181a in CSF is a potential auxiliary biomarker for identifying occult CNS leukemia, addressing a long-standing diagnostic challenge.

Circulating miRNAs may also predict therapy response and relapse. Because miRNAs often modulate pathways of cell proliferation and drug resistance, their levels can reflect how the leukemia will behave. As an example, miR-326 has been implicated in drug resistance in ALL. Saffari et al. (2024) examined *exosome-associated* miR-326 (discussed more under EVs) and found higher exosomal miR-326 in ALL patients who were chemoresistant, but interestingly this miRNA acted as a tumor suppressor by reducing leukemic cell viability (3). This suggests that low levels of certain miRNAs might mark high-risk disease, whereas restoring those miRNAs could be therapeutic. Another report noted that upregulation of miR-125b and miR-99a in plasma was associated with early treatment response in pediatric ALL (26) (though data are still emerging). Overall, a variety of miRNA profiles have been proposed to either distinguish ALL subtypes or forecast outcomes, but there is not yet a single consensus miRNA panel in clinical use.

Technologically, measuring circulating miRNAs requires careful normalization and control. One challenge is that different patients (and even different sample handling protocols) can have widely varying baseline miRNA levels. Unlike DNA, which is more straightforwardly present or absent, RNA markers demand robust reference controls to account for factors like hemolysis (which can release miRNAs from blood cells). Researchers are working to identify stable reference miRNAs or small RNAs for data normalization in leukemia studies (16, 27). Another challenge is specificity: changes in some circulating miRNAs (e.g. those involved in inflammation or cell death) may not be unique to leukemia. Nevertheless, miRNA profiling offers a complementary layer of information. While ctDNA focuses on genetic mutations, miRNAs reflect regulatory network changes induced by the leukemia in a direct or indirect fashion. The two together can provide a more holistic picture. A future vision is that a clinician might use a *miRNA panel* as an initial screening tool (since it’s relatively simple and cheap to do by PCR) to flag potential ALL or gauge risk, and use ctDNA sequencing for detailed genotyping and MRD quantification.

Circulating miRNAs are highly vulnerable to pre-analytical interference such as hemolysis, freeze-thaw cycles and inconsistent centrifugation. They are also secreted by multiple normal cells, resulting in non-specific background noise. At present, miRNA panels lack unified detection standards and large-scale prospective validation, and are not ready for routine clinical use (16, 28).

validation, and are not ready for routine clinicalIn conclusion, circulating miRNAs demonstrate potential promise as non-invasive biomarkers in pediatric ALL, particularly for aiding diagnosis and potentially identifying high-risk disease or CNS involvement. Ongoing studies and clinical trials are expected to validate specific miRNAs or signatures. If successful, with further validation, blood miRNA tests may potentially be integrated into routine diagnostic workflows alongside conventional assays – for example, a high-risk miRNA profile at diagnosis might prompt closer monitoring or a different therapeutic approach. Before clinical adoption, however, issues of assay standardization, reproducibility, and large-cohort validation must be addressed. Circulating miRNA studies are mostly limited to small-sample single-center research, lacking unified standards and large-scale prospective verification.

## 5 Extracellular vesicles and exosomes in ALL

Extracellular vesicles (EVs) are membrane-bound particles released by cells into bodily fluids. They include exosomes (30–200 nm nanovesicles originating from endosomal compartments) and microvesicles shed from the cell surface. EVs carry a cargo of biomolecules derived from their parent cells – proteins, lipids, DNA, various RNAs (mRNA, miRNA, lncRNA, etc.) – protected by a lipid bilayer (3). In cancer, tumor-derived EVs can facilitate intercellular communication, modulate the microenvironment, and even prepare metastatic niches. In pediatric ALL, EV research is still nascent but rapidly growing, as EVs represent a rich trove of potential biomarkers and therapeutic targets (27–30).

Leukemia cells secrete exosomes that have systemic effects. A striking example is their impact on the immune system. Gholipour et al. studied exosomes isolated from the serum of children with pre-B ALL and co-cultured them with healthy T cells (31). They observed that the ALL-derived exosomes had immunosuppressive effects: they induced T-cell apoptosis and skewed T-cells toward a regulatory (T-reg) phenotype by upregulating FOXP3 and IL-10, while reducing pro-inflammatory Th17 signals (31). This suggests that ALL exosomes help the leukemia evade immune surveillance. The presence of such immunosuppressive exosomal cargo could serve as a biomarker of disease burden or progression – essentially indicating that the tumor is actively remodeling the immune environment. They propose that these exosomal signals might correlate with disease “staging” or risk, as more aggressive disease could release more EVs that suppress immunity (31).

From a biomarker standpoint, the contents of EVs can be profiled to reflect the leukemia. One area of interest is exosomal microRNAs (a cross-over with the previous section). Because many circulating miRNAs are actually enclosed in exosomes, some studies specifically analyze exosome-derived RNA. For instance, the above-mentioned miR-326 was studied in the context of exosomes: researchers isolated plasma exosomes from drug-resistant vs drug-sensitive pediatric ALL patients. They found miR-326 levels significantly higher in exosomes from ALL patients than controls (AUC ~0.75 for distinguishing ALL) and even higher in those with drug-resistant disease, suggesting its potential as a drug-resistance associated biomarker (3). Fascinatingly, independent of this clinical correlation, these miR-326-rich exosomes could transfer their content to recipient leukemia cells and exert intrinsic anti-leukemic effects by reducing the viability of drug-resistant cells. Current available data cannot fully exclude synergistic effects from other functional cargos within exosomes; it remains unclear whether the reduced cell viability arises from independent miR-326 activity or combined impacts of multiple vesicle-contained biomolecules. In other words, exosomes were acting as vehicles of a tumor-suppressive miRNA, hinting at a potential therapeutic angle: using exosomes or mimics to deliver miR-326 to resistant leukemia cells might overcome resistance (3). From a prognostic perspective, measuring exosomal miR-326 in newly diagnosed patients could identify those predisposed to drug resistance (so-called primary resistant cases) (3). Another study in 2025 suggests a complex network where lncRNA HOTAIR in leukemia cells influences which miRNAs (like 326) get packed into exosomes (32). Such mechanistic insights are beyond our scope, but they reinforce that EV cargo selection is biologically meaningful.

Beyond RNA, exosomal DNA and protein are also being explored. Tumor-derived exosomes can contain double-stranded DNA fragments spanning the genome, which in principle could be sequenced to identify leukemia-specific mutations (similar to ctDNA). It remains an intriguing possibility for future research. On the proteomics side, leukemia exosomes might carry characteristic surface proteins or internal enzymes that could serve as markers. For example, an analysis of EV surface proteins could potentially detect ALL-specific antigens or differentiate B-ALL vs T-ALL EVs. A 2021 analytical study developed a SERS (surface-enhanced Raman scattering) method combined with machine learning to classify cancer EVs, which in principle could be applied to ALL (33).

Clinically, EVs hold appeal not only for diagnostics but also as therapeutic agents or targets. As mentioned, exosomes could potentially be engineered to carry anti-leukemic signals (like miR-326 or drugs) and deliver them to sites like the bone marrow or CNS sanctuary. Conversely, blocking the release or uptake of leukemia EVs might mitigate their harmful effects (such as immune suppression or drug resistance promotion). However, these ideas are still far from clinical translation and require much more understanding of EV biology in ALL.

From a diagnostic perspective, EV-based assays are not yet routine due to several practical challenges. Isolating exosomes from blood typically requires ultracentrifugation or specialized kits, which can be time-consuming and yield variable results depending on technique (34). Standardization is difficult – the field is still working on consistent EV isolation and characterization methods (e.g., the ISEV guidelines) (26). Pediatric samples, being limited in volume, complicate large-scale EV analysis, since yields of exosomes are proportional to starting volume. Nonetheless, even small plasma volumes (1-2 mL) can contain millions of exosomes, so microfluidic and immunoaffinity methods might enable analysis from finger-prick blood in the future. Another hurdle is specificity: blood contains EVs from many sources (platelets, immune cells, etc.), and leukemia EVs must be distinguished from this background. Selecting markers unique to leukemia EVs (perhaps certain RNA or protein combinations) is an active area of research (34).

Extracellular vesicles represent an exciting frontier in pediatric ALL liquid biopsy. They offer a window into cell–cell communication in leukemia and carry multi-modal biomarkers (RNA, DNA, protein) in a stable form. Early studies demonstrate that ALL exosomes can modulate the immune system and that their contents (like miR-326) have diagnostic and prognostic relevance. While technical challenges remain, the ongoing improvements in EV isolation and analysis are likely to make EV-based biomarkers increasingly feasible. We can anticipate that in the coming years, an EV profile (for example, a set of exosomal miRNAs or proteins) could be integrated with ctDNA and clinical data to refine risk stratification in pediatric ALL. EV-related research is still in the early exploratory stage with poor technical standardization and insufficient pediatric ALL-specific validation data. Apart from expensive detection equipment, repeated reagent consumption and dependence on professionally trained testing personnel further restrict large-scale routine adoption of high-sensitivity liquid biopsy assays in under-resourced pediatric medical centers worldwide.

## 6 Clinical applications and challenges

1. Diagnostic use: Liquid biopsy tools are gradually supplementing conventional diagnostic methods for ALL. While a bone marrow aspirate is still required to definitively diagnose ALL (to enumerate blasts and perform comprehensive phenotyping (3)), liquid biopsy can aid in identifying molecular markers at diagnosis in a less invasive manner. For instance, a blood sample could be used to detect ALL-specific genetic lesions (like translocations or copy number changes) even before a marrow result is available (6). In the future, one could envision a diagnostic workflow where, upon suspicion of leukemia, a “liquid biopsy panel” is run on blood to screen for common ALL-associated mutations/fusions and miRNA profiles – if positive, this could prompt urgent marrow examination and also guide initial risk stratification. Additionally, for patients where marrow aspiration is contraindicated or delayed, a ctDNA analysis might provide critical information (e.g. the presence of a high-risk genotype like *KMT2A* rearrangement) to guide therapy initiation.
2. Prognosis and risk stratification: Liquid biopsy markers have demonstrated correlations with known risk factors and outcomes. High levels of certain miRNAs (e.g. miR-146a, miR-155) or ctDNA persistence after induction therapy have been associated with higher risk of treatment failure (2, 35). Mechanistically, these dysregulated miRNAs are not just passive biomarkers. Rios de Los Rios et al. showed that B-ALL cells secrete exosomal miRNAs that act as TLR8 agonists, inducing a proinflammatory bone marrow microenvironment and promoting treatment resistance (36). Conversely, clearance of circulating markers mirrors good response. A recent study showed that patients with larger drops in cfDNA concentration early in therapy had significantly better event-free survival. Some liquid biopsy findings might refine risk groups – for example, a standard-risk ALL patient (by traditional criteria) who has detectable ctDNA MRD after induction might be upgraded to high-risk for therapy intensification. It is worth noting that adult studies in AML and lymphoma have already established that ctDNA MRD is an independent prognostic factor for relapse (37). Pediatric ALL is following suit, with several trials now incorporating exploratory ctDNA/MRD endpoints. Eventually, the hope is to integrate liquid biopsy results into MRD-based risk algorithms, to improve their predictive power.
3. MRD monitoring: Perhaps the most impactful application of liquid biopsy in ALL is measurable residual disease monitoring. MRD is the cornerstone of modern ALL management – patients with MRD negativity after induction have far better outcomes than those with MRD positivity (38). Currently, MRD is assessed via bone marrow aspirate (flow cytometry or PCR). Liquid biopsy offers a way to monitor MRD more frequently and non-invasively in blood. The evidence, as reviewed, indicates that plasma ctDNA can detect MRD with high sensitivity and even catch residual disease that escapes detection in marrow. This could be especially useful during maintenance therapy or post-therapy follow-up, where routine marrow biopsies are avoided but the risk of relapse still exists. For example, instead of waiting for hematological relapse (overt rise in blasts) or performing periodic marrows, clinicians could perform monthly blood ctDNA tests. A conversion from negative to positive ctDNA could signal a molecular relapse and trigger early intervention, potentially preempting a full-blown relapse. This approach has already shown success in other malignancies like lymphoma, and pediatric ALL researchers are actively investigating it. However, to implement this clinically, standardized assays (e.g., a CLIA-certified NGS panel for ALL ctDNA) and defined thresholds for positivity are required. Another challenge is ensuring specificity – low-level ctDNA might theoretically come from clonal hematopoiesis (aging-related mutations in blood cells) rather than leukemia, though this is less common in children.
4. Relapse prediction and detection: Beyond MRD shortly after therapy, liquid biopsy can be used during remission surveillance. Studies in adult AML have shown ctDNA can predict relapse months in advance of symptoms or blood count changes (39). In pediatric ALL, anecdotal evidence and early studies suggest a similar trend. For instance, rising levels of ALL-specific DNA (such as an IGH clone or fusion gene) in the blood could herald relapse, even if the child is clinically well. Likewise, a trend of increasing oncogenic miRNAs or returning abnormal EV signals might indicate that minimal disease is expanding. Early relapse detection gives a critical window for action – potentially starting salvage therapy when the tumor burden is low, which might improve outcomes. It also facilitates monitoring the effectiveness of novel interventions (e.g. if immunotherapy is given in a molecular relapse, one can track ctDNA to see if it clears). The limitation is that not all relapses shed enough ctDNA at very low disease burden, so a negative result does not guarantee absence of disease. A combination of marrow checks (at longer intervals) and interim liquid biopsies might strike the right balance.
5. Pediatric-specific challenges: Applying liquid biopsy in children comes with some unique hurdles. One is the constraint of blood volume – pediatric patients, especially infants or small children, cannot safely provide large blood samples. Fortunately, modern ctDNA assays can work with a few milliliters of plasma; in fact, as little as 1 mL of plasma can be sufficient for ctDNA-based tumor monitoring in pediatric patients (9). Nonetheless, if serial sampling is needed, careful planning is required to stay within phlebotomy limits. Assay sensitivity is paramount given the often tiny tumor burden after treatment. The low ctDNA fraction demands techniques that push the limits of detection (digital PCR, error-corrected NGS). This high sensitivity raises issues of false positives as well – an erroneous call of MRD could subject a child to more therapy, so assays must be meticulously validated. Another pediatric consideration is the biological differences in tumors: ALL in children has distinct genetic features (like high incidence of certain translocations or hyperdiploidy) compared to adult leukemias (40). Liquid biopsy assays must encompass the full range of pediatric ALL alterations. For example, a commercial panel designed for adult leukemia might miss a pediatric-specific alteration; thus bespoke design or pediatric-focused panels may be needed. Circulating cfDNA kinetics vary with age among children, and ultra-low-volume blood samples require specially optimized detection protocols. In addition, disease characteristics and biomarker performance in infant ALL also deserve independent attention in clinical practice. Nevertheless, high-precision sequencing and digital PCR platforms bring additional equipment and economic burdens, limiting the widespread accessibility of liquid biopsy in low-resource medical settings.
6. Standardization and validation: Most published liquid biopsy studies in pediatric ALL are single-center investigations with relatively small cohorts, varying biomarker selections and analytical platforms, and lack unified large-scale prospective validation standards (41). A recurring theme for liquid biopsy (in ALL and beyond) is the need for standardization. Pre-analytical variables like how blood is collected, processed, and stored can greatly affect results (e.g., delays in plasma separation can increase background cfDNA) (42). Efforts are underway to define standard protocols for handling pediatric liquid biopsy samples. Similarly, different labs may use different PCR primers or sequencing methods – harmonizing these and establishing quality control measures will be important so that results are comparable across centers. Large-scale validation in clinical trials is the path to acceptance. Encouragingly, there are ongoing trials and consortium studies focusing on liquid biopsies in childhood cancers (43). As data accumulate, we will better understand how to interpret liquid biopsy results in ALL – for instance, what level of ctDNA corresponds to a 10^−4^ MRD by flow, or how a specific miRNA change translates to risk. Regulatory approval will follow once clinical utility is proven. The FDA has already approved some blood-based NGS tests for solid tumor biomarkers (44), and in hematology the first applications (like clonoSEQ for tracking lymphoid malignancy clones) are making their way into practice (45, 46). It is plausible that within the next decade, an FDA-approved ctDNA MRD test for pediatric ALL will become available if current research is successful.
7. Ethical considerations: Adopting liquid biopsy in pediatric ALL also raises ethical and practical questions. On one hand, reducing invasive procedures aligns perfectly with pediatric ethics – minimizing harm and stress to young patients. Replacing a bone marrow puncture with a blood draw is a clear win for quality of life and safety. On the other hand, one must ensure that any new test is adequately validated so as not to inadvertently harm patients through false results. In a research context, families should be counseled on what will be done with liquid biopsy findings. For example, if an experimental ctDNA test suggests an impending relapse, but standard clinical criteria are not met, should treatment be altered? This touches on the ethics of acting on unvalidated tests. Ideally, such situations are handled within clinical trials that have predefined rules. In addition, there are considerations of privacy and consent: genomic sequencing of ctDNA could reveal incidental genetic information (including germline variants or predispositions) that families may or may not want to know. Ensuring proper informed consent and counseling for such possibilities is important when performing comprehensive NGS-based liquid biopsies. Another aspect is equity and access – advanced molecular tests can be expensive, so there is an ethical imperative to make sure these innovations benefit all patients and do not widen outcome disparities. Advocacy and research in the public sector (and support from initiatives like pediatric oncology consortia) can help in this regard, so that liquid biopsy becomes an accessible standard tool, not a boutique test available only at elite centers.

## 7 Conclusion and future directions

The future of liquid biopsy in pediatric ALL is bright. We anticipate increasingly *multimodal* approaches – combining ctDNA, circulating RNA, and EV analyses to capture complementary information about the leukemia. Machine learning models might integrate these data with clinical variables to improve prediction of outcomes (for example, AI algorithms to stratify risk based on longitudinal ctDNA trends). With targeted and immunotherapies now central to pediatric ALL precision care (47, 48), liquid biopsy serves as a cornerstone for real-time monitoring, MRD detection, and personalized therapy guidance, aiming to boost survival, minimize toxicity, and enhance children’s quality of life (49). Building on this, liquid biopsy could also guide truly personalized therapy: one can monitor in real-time which clones are expanding and tailor drugs accordingly, or detect emerging resistance mutations and switch therapy before relapse fully develops. Moreover, liquid biopsy might enable new endpoints in clinical trials (such as molecular response rate, instead of waiting for morphological response). As assays become cheaper and faster, even point-of-care testing is conceivable – e.g., a rapid cartridge-based PCR test for an ALL-specific DNA sequence from a fingerstick blood sample. Finally, lessons from adult oncology will continue to inform pediatric use. The field acknowledges that pediatric liquid biopsy efforts are “still behind adult oncology”, but publications and innovations in pediatrics have sharply increased in the last few years. With ongoing validation in pediatric trials and growing clinician familiarity, liquid biopsy is moving from bench to bedside.

In conclusion, liquid biopsy in pediatric ALL is a transformative approach that complements traditional methods of disease assessment. By enabling non-invasive, sensitive detection of tumor-derived biomarkers like ctDNA, miRNAs, and exosomes, it holds the potential to improve diagnostic accuracy, refine prognostication, and allow earlier intervention for relapse – all while reducing the burden on young patients. Challenges of sensitivity, standardization, and interpretation are actively being addressed through research. As evidence mounts, it is likely that the coming years will see liquid biopsy assays integrated into routine pediatric ALL management, heralding a new era of precision monitoring and truly individualized therapy for children with leukemia.
